# Assessing ecological uncertainty and simulation model sensitivity to evaluate an invasive plant species’ potential impacts to the landscape

**DOI:** 10.1038/s41598-020-75325-w

**Published:** 2020-11-04

**Authors:** Catherine S. Jarnevich, Nicholas E. Young, Catherine Cullinane Thomas, Perry Grissom, Dana Backer, Leonardo Frid

**Affiliations:** 1 U.S. Geological Survey, Fort Collins Science Center, 2150 Centre Ave Bldg C, Fort Collins, CO 80526 USA; 2 Natural Resource Ecology Laboratory, Colorado State University, Fort Collins, CO 80523-1499 USA; 3 Saguaro National Park, 3693 South Old Spanish Trail, Tucson, AZ 85730 USA; 4 Apex Resource Management Solutions Ltd, 937 Kingsmere Avenue, Ottawa, ON K2A 3K2 Canada; 5 Present Address: Coronado National Forest, Tucson, AZ 85701 USA

**Keywords:** Invasive species, Ecological modelling

## Abstract

Ecological forecasts of the extent and impacts of invasive species can inform conservation management decisions. Such forecasts are hampered by ecological uncertainties associated with non-analog conditions resulting from the introduction of an invader to an ecosystem. We developed a state-and-transition simulation model tied to a fire behavior model to simulate the spread of buffelgrass (*Cenchrus ciliaris*) in Saguaro National Park, AZ, USA over a 30-year period. The simulation models forecast the potential extent and impact of a buffelgrass invasion including size and frequency of fire events and displacement of saguaro cacti and other native species. Using simulation models allowed us to evaluate how model uncertainties affected forecasted landscape outcomes. We compared scenarios covering a range of parameter uncertainties including model initialization (landscape susceptibility to invasion) and expert-identified ecological uncertainties (buffelgrass patch infill rates and precipitation). Our simulations showed substantial differences in the amount of buffelgrass on the landscape and the size and frequency of fires for dry years with slow patch infill scenarios compared to wet years with fast patch infill scenarios. We identified uncertainty in buffelgrass patch infill rates as a key area for research to improve forecasts. Our approach could be used to investigate novel processes in other invaded systems.

## Introduction

Invasive species are non-native species that cause harm to, or negatively impact, the system to which they are introduced. These impacts can vary widely, and the impacts are generally positively related to the abundance of the invasive species^1^. Impacts can occur at different scales within an ecosystem, including impacts to individual organisms, population genetics, population dynamics, communities, and ecosystem processes^2^. Effects on ecosystem properties can include wildfire frequency and extent, such as those found with invasive grass species in the western United States^3^. Furthermore, invasive species can respond to and interact with changing environments in ways that shift or modify the magnitude of their impact^4^. Forecasts of invasive species’ impacts are needed to inform both risk assessments and to prioritize management actions^5^. Understanding the potential geographic extent of an invasive species across a landscape and associated impacts to native systems can help define the degree of the problem.

Invasive species, like native species, can be influenced by many interacting abiotic and biotic factors. These factors, including climate, can influence rates of spread across a landscape and patch infilling in established locations, adding variability to invasion dynamics (e.g.,^6^). A patch can be defined as the perimeter of an infested area, and infilling is the increase in cover within that defined patch. Spread occurs when patch perimeters expand or when new satellite patches establish. Spread and infill, particularly in the arid southwestern United States, may be tied to fluctuating resources such as interannual variation in precipitation^7^. Patch infilling is likely tied to the number of seeds produced by a patch and seedling germination and survival, which can be influenced by climate^8^.

There are many types of models that can be used to evaluate ecological change. Spatially explicit models include, among others, invasive species spread models, individual based models, network models, or potential distribution models. There are also aspatial models such as vegetation dynamics models used to quantify vegetation class amounts within a landscape. Area- or state- based models that inform how the condition or composition of a piece of land may change over time can provide key information to managers who are concerned with the future condition of a landscape.

State-and-transition simulation models (STSM) are an increasingly employed area-based simulation tool used to simulate landscape dynamics and evaluate complex and often uncertain future conditions^9^ and have been used to forecast plant invasions^10–13^. STSMs divide a landscape into discrete spatial units (cells) each assigned a discrete state; simulations track changes in cell state over discrete time steps based on discrete transitions between states that can be probabilistic or time-based and can be unique for each transition type (e.g., succession, wildfire, timber harvest, herbicide spraying). STSMs, and other complex models representing ecosystem processes, require multiple parameters that may be stochastic and uncertain. Uncertainties in ecological forecasts using STSMs can include ecological process variables and environmental stochasticity (e.g.^14^). One utility of STSMs for invasive species is identifying research priorities to explore uncertainties that have a strong influence on modeled outcomes, along with helping to identify ‘how big is the problem?’ and appropriateness of various management strategies^15^. Here we investigate the effects of model uncertainties on the simulated spread and infill of invasive buffelgrass (*Cenchrus ciliaris* L. syn *Pennisetum ciliare* (L.) Link) in Saguaro National Park (SAGU), located within the Sonoran Desert in southern Arizona, USA (Supplementary Figure S1 online).

Buffelgrass is an invasive perennial grass native to Africa that has been introduced widely as a forage species and for erosion control^16^. Following its introduction, buffelgrass has spread and become invasive in many natural areas globally where it has various impacts on native ecosystems. Various species distribution models exist for buffelgrass, including global and regional models under current and future climate that provide information on its potential distribution^17–19^. In the Sonoran Desert, conversion of native vegetation to buffelgrass savanna has altered ecohydrology^20^, altered fire regimes^21,22^, and negatively impacted populations of native species^23–25^. Altered fire regimes and impacts to native populations have also been observed in Australia^26–28^.

Experts on the buffelgrass invasion in southern Arizona have identified vegetation conversion, fire, and the loss of biodiversity and native species, especially the loss of saguaro cacti (*Carnegiea gigantea* (Engelm.) Britton & Rose), as the primary threats caused by the buffelgrass invasion in the Sonoran Desert^29^. The introduction of buffelgrass to the Sonoran Desert, which includes many non-fire adapted species, leaves managers with a high degree of uncertainty about the trajectory of the invasion and associated landscape changes because no analog ecological conditions exist that would allow them to make more than broad, general predictions^30^. There is a critical need to understand the nature of the threat posed to native ecosystems by this invasive species to effectively allocate control efforts and ensure long-term land management goals are met. Misjudging the dynamics of the situation may waste scarce resources and allow priority resources or locations to be adversely and irreversibly impacted.

Buffelgrass growth and vigor in desert ecosystems can be influenced by the amount of precipitation during a growing season^31,32^, and the effect of variable precipitation on buffelgrass infill rates is a primary uncertainty identified by local experts^29^. The southwest region has exhibited wide annual variation in monsoon season precipitation^33^, with the late 1970s and 1980s being relatively wet compared to past and current decades^34^. Previous simulation modeling to evaluate the buffelgrass problem in Saguaro National Park did not include an evaluation of the sensitivity of the simulations to model initialization or to uncertainties in model parameterization, limiting the ability of simulations to capture the range of potential future landscape conditions^29^. Model initialization describes landscape starting conditions, including landscape susceptibility to buffelgrass invasions and where and how much buffelgrass is on the landscape. Uncertainties in model parameterization include uncertainty in patch infill rates and uncertainty in how these rates may vary with precipitation^29^.

Our objectives were to forecast the potential influence of buffelgrass on the Saguaro National Park landscape, including loss of native desert vegetation and altered fire dynamics given ecological uncertainties, and to identify which uncertainties most influence forecasts and thus may have the largest impact on management decisions. We examined the sensitivity of STSM results to model initialization and to ecological uncertainties across an expert-identified range of possible values^29^ along with uncertainty related to stochasticity between individual model runs. We explored the potential impacts from buffelgrass competition alone versus impacts of an altered disturbance regime (i.e., fire). We considered two groups of uncertainties: those associated with underlying landscape characteristics at the start of the simulation (model initialization scenarios) and those associated with model parameterization including infill rates and the influence of precipitation on infill rates (ecological scenarios). The simulations were run in the absence of management actions and highlight the potential range in the magnitude of the invasion if buffelgrass is left untreated—an important metric requested by regional experts.

## Methods

### Study area

Saguaro National Park (here forth referred to as SAGU), located in the Sonoran Desert in southern Arizona, is divided into two units separated by the city of Tucson (Supplementary Figure S1 online). The 27,279 ha Rincon Mountain District (RMD) located to the east of Tucson has a greater elevational difference and includes a desert ecosystem, grassland ecotone, and high-elevation forest. The 9726 ha Tucson Mountain District (TMD) located to the west of Tucson is smaller, sits at a lower elevation, and includes only the desert ecosystem. Despite the geographic separation the two units are managed as a whole, so we modeled the two districts together in our simulations. Climate, recorded by the Tucson International Airport Weather Station (period of record 6/1/1946 to 6/9/2016 from Western Regional Climate Center), is arid/ semi-arid characterized by monsoonal precipitation (average of 29 cm/year) with an average January minimum temperature of 3.7 °C and average July maximum temperature of 37.4 °C.

### STSM model

This research used a previously developed state-and-transition simulation model for buffelgrass in Saguaro National Park^29^. The model divides the landscape into three strata in a 0.25-ha resolution grid, including the desert ecosystem, classified into regions that are susceptible or unsusceptible to buffelgrass establishment (based on buffelgrass habitat suitability^17^), and the grassland ecotone between the desert ecosystem and high-elevation forest that exists in RMD (forested areas of SAGU are not included in the study area; Supplementary Figure S1 online). Susceptible locations were defined as locations predicted as suitable for buffelgrass by a habitat suitability model developed for SAGU using their buffelgrass location data and environmental characteristics including winter temperature, slope, northness and eastness^17^. The desert ecosystem not classified as susceptible was assigned to unsusceptible. The STSM also assigns each grid cell of the landscape to state classes related to buffelgrass cover including uninvaded, seedbank (cells with buffelgrass seeds but no established buffelgrass), buffelgrass cover classes of < 1%, 1–10%, > 10–50%, and > 50%, and a converted state consisting of a buffelgrass savanna as a result of burning. Operationally, each grid cell can be thought of as a patch, and infilling is the increasing cover of buffelgrass within that cell. Landscape state classes also correspond to vegetation fuel load to facilitate use of a fire behavior model. Greater than 10% buffelgrass cover is required to carry fire and fire in a > 50% cover class results in a converted state^29^.

**Figure 1:**
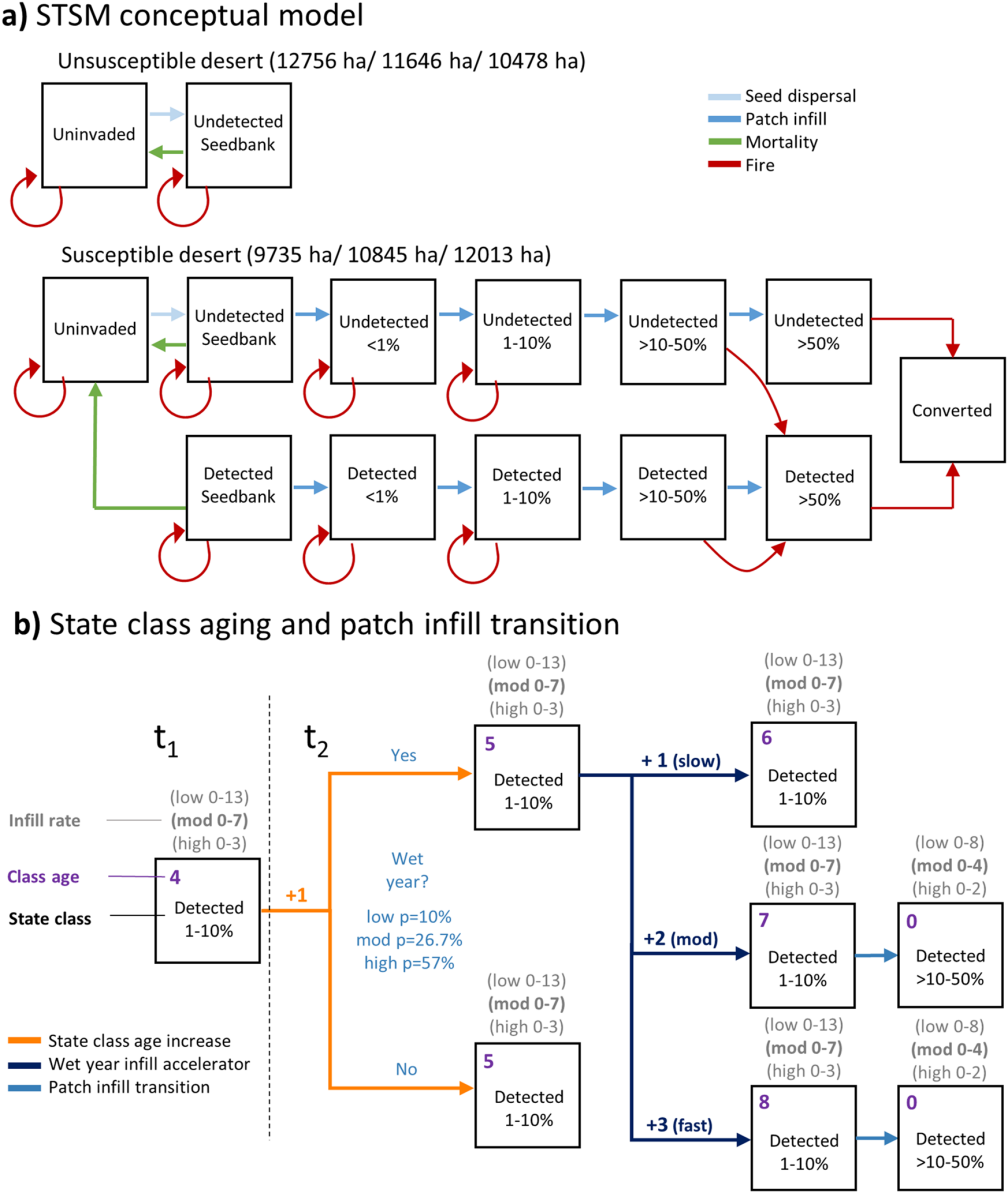
**(a) **Conceptual state and transition model (STSM) for two strata, unsusceptible desert and susceptible desert, modified from Jarnevich et al. ^29^ to exclude management-induced ecological transitions and retain transitions related to seed dispersal, patch infill, seedbank mortality, and fire. The three areas (in parentheses) for each stratum include the area of the landscape in unsusceptible and susceptible desert, respectively, based on habitat suitability thresholds of low, moderate, and high susceptibility to buffelgrass establishment. Note that in scenarios without fire, the fire-induced ecological transitions were not included. Percent of detected and undetected buffelgrass cover is shown. (**b**) Flowchart demonstrating state class aging and patch infill transition between two time steps (t1 and t2). The parameters that influence patch aging and cover-class transition are the infill rate (i.e., the time within a given cover class), the probability of a wet-year, and the wet-year patch infill acceleration factor. This example uses the moderate infill rate (in bold, 0–7 represents number of years spent in the cover class) and demonstrates multiple paths depending on if the year is a wet-year, and if wet, the wet-year infill acceleration factor scenario (slow, moderate, or fast). The patch infill transition occurs only when the infill rate reaches the maximum age for the given cover class, otherwise the patch infill transition does not occur and only the class age increases.

Because we were specifically interested in investigating the impacts of ecological uncertainty on buffelgrass forecasts in SAGU, we used a modified version of the Jarnevich et al.^29^ STSM that eliminated management-induced ecological transitions between state classes, retaining all ecological transitions including seed dispersal, buffelgrass cover increases, seedbank mortality, and fire-induced ecological transitions (Fig. 1a). Because we excluded management actions, we further modified the original STSM by collapsing the detected and undetected classes that describe whether a cell is known to be invaded or not into states distinguished only by cover class (e.g., ‘detected < 1% cover’ and ‘undetected < 1% cover’ both became ‘ < 1% cover’).

### Model initialization

We conducted sensitivity analyses on a key uncertainty in model initialization: the total amount of SAGU defined as being susceptible to buffelgrass establishment. Each grid cell of the desert ecosystem was assigned as susceptible or unsusceptible based on a habitat suitability model developed for SAGU^17^. The habitat suitability model produces continuous values between 0 and 1, and a threshold value is selected to discretize the predictions into binary suitable or unsuitable classes. To test the sensitivity to the habitat suitability threshold decision, we varied threshold value between 0.45 and 0.55, which resulted in different amounts of grid cells assigned to the susceptible strata. These values increase (0.45) and decrease (0.55) the estimated amount of susceptible area on the landscape by roughly ± 10% relative to the original index used for simulations (0.50) that was determined based on the habitat suitability model evaluation^17^. We refer to the threshold value of 0.45 as high (more susceptible area), 0.50 as moderate, and 0.55 as low (less susceptible area; Table 1).

**Table 1:** Values used in sensitivity analysis of model initialization for Saguaro National Park including amount of susceptible habitat and the amount of the landscape that was initially assigned to each buffelgrass cover class in hectares.

	Low initialization	Moderate initialization	High initialization
Susceptible habitat	9735	10,845	12,013
< 1% cover	173.9	178.8	184
1–10% cover	807	818.3	829.5
> 10–50% cover	503	503	503
> 50% cover	121.8	121.8	121.9

The amount of buffelgrass on the landscape at the start of the simulations was assigned based on SAGU mapping data. In addition to known buffelgrass locations, we randomly assigned some cells to buffelgrass state classes based on detection probabilities estimated by SAGU staff^29^. Buffelgrass occurrence at SAGU is regularly inventoried and SAGU staff and the authors felt that their estimate of undetected buffelgrass could reasonably be assumed to be within ± 10% of the true value of undetected cells for the entire SAGU. To account for uncertainty in the amount of undetected buffelgrass present on the landscape, we varied the amount of buffelgrass at the start of the simulation by either increasing the number of grid cells assigned to buffelgrass cover classes by 10% (high) or decreasing the number by 10% (low). This resulted in very small changes in the overall amount of buffelgrass present at model initialization (Table 1).

### Model parameterization

Regional experts defined the transition between buffelgrass cover classes as deterministic based on amount of time spent in a state class, but expressed uncertainty related to the amount of time required in each state before transitioning. We elicited values from regional experts related to the time required before transitioning to the next buffelgrass cover class including lower-quartile, median, and upper-quantile estimates for each transition time^29^. We refer to these transition times as the patch infill rate.

Regional experts also indicated that patch infill rates could be influenced by precipitation amounts during the monsoon season. This influence could be broken into two pieces: the probability that a year is ‘wet’, and a patch infill acceleration factor resulting from greater precipitation in a ‘wet’ year (hereafter referred to as wet-year infill acceleration factor). To determine the probability of a ‘wet’ year, we examined historic climate variability to calculate the probability of having a year with above median monsoon length and above median cumulative monsoon precipitation. We compared individual years from 1987 to 2016 to a 30-year median of monsoon length and cumulative monsoon precipitation using data from the nearest long term weather station, located at Tucson International Airport (GHCND:USW00023160; obtained from www.ncdc.noaa.gov on 23 Feb 2017). This comparison resulted in 26.7% of years with values above the 30-year median. We also calculated the lower-quartile (10% of years) and the upper-quartile (57% of years). We randomly assigned years as being ‘wet’ within the simulations based on these probabilities using the random number function in R version 3.4.2^35^. This process was repeated for 20 Monte Carlo realizations per model scenario except for the scenarios that included fire simulations (described below).

The wet-year infill acceleration factor decreases the time to deterministic transitions between cover classes during ‘wet’ years^29^. This effect was elicited as a divisor that would divide the total time needed to be spent within an age class, and thus described the patch infill acceleration factor that would occur if all years were ‘wet.’ We refer to the elicited lower quantile value as ‘slow’, average value as ‘moderate’, and upper quantile value as ‘fast’. The simulation model tracks the amount of time spent within a state class per cell, typically adding one year to the class age of the cell at each time step. To implement the acceleration factor on a year-by-year basis in the model, we converted the divisor to an additive effect such that the elicited divisors of 2, 3, and 4 (rounded to whole numbers) resulted in transitions occurring 2 × , 3 × , and 4 × as quickly. Translating this to a single year time-step meant that a 2 × transition would add 1 additional year to the age of a cell (1 default + 1 acceleration factor = 2 years), a 3 × transition would add 2 additional years to the age of a cell (1 default + 2 acceleration factor = 3 years), and a 4 × transition would add 3 additional years to the age of a cell (1 default + 3 acceleration factor = 4 years). Generalizing this pattern, a wet-year divisor of n is implemented within a timestep by adding the default 1 year + (n – 1) additional years to the cell age during a ‘wet’ year, thus decreasing the overall time spent within a cover class (Fig. 1b).

### Model simulations

We implemented the STSM described above using the stsim base package (version 3.1.21^9^) with the stsim-farsite add-on package (version 3.1.21) running on SyncroSim version 2.0.41. The stsim-farsite package integrates the FARSITE fire area simulator software version 4.1.055^36^ into ST-Sim. We provided FARSITE with current time step fuel model attributes of each cell in the landscape along with the number of ignitions (one per time step) and weather information (see^37^ for details), and FARSITE then produced fire perimeters at each time step. If ignitions occurred in non-burnable areas, the perimeter would be zero for that time step.

We ran simulations both with and without fire. We ran each simulation for 30-time steps (from 2014–2044), including 20 Monte Carlo realizations to capture stochasticity in the ecological processes. We considered uncertainties associated with model initialization (initialization scenarios) and uncertainties associated with model parameterization (ecological scenarios). Initialization scenarios included each combination of habitat suitability and initial conditions (nine initialization scenarios). Ecological scenarios included combinations of all three factors affecting cover-class transition rates (infill rate, wet-year probability, and wet-year infill acceleration factor; for a total of 27 ecological scenarios). We were interested in the interaction of these uncertainties with fire on the landscape; however, including FARSITE at each timestep to simulate fire is much more computationally intensive than simulation runs without fire. For this reason, we ran all 27 ecological scenarios without fire, and only ran a subset of ecological scenarios with fire. For fire simulations we ran three combinations of ecological parameter levels: the lower bounds (low infill rate, low wet-year probability, slow infill acceleration factor—referred to as dry-slow patch infill), the middle bounds (moderate infill rate, moderate wet-year probability, moderate wet-year infill acceleration factor—referred to as moderate patch infill), and the upper bounds (high infill rate, high wet year probability, fast wet year infill acceleration factor—referred to as wet-fast patch infill).

Due to computational limitations, the timing of ‘wet’ years was not varied for fire simulations across the 20 Monte Carlo realizations, while the timing of no-fire simulations was varied across iterations as described above. To investigate the impact of constant versus variable timing of ‘wet’ years on model outcomes, we also ran the no-fire simulations with the same constant ‘wet’ years across iterations as those used for the fire simulations (Supplementary Figure S2 online).

We analyzed invaded area through time, calculated by multiplying the area occupied by each cover class by the midpoint percent cover for that class (i.e., multiplied area in < 1% cover by 0.005, 1–10% by 0.055, > 10–50% by 0.3, > 50% by 0.75, and converted by 1.0), and compared invaded area at the end of simulations across scenarios. We also compared the amount of the landscape in each state class at the start and end of the simulations (i.e., after 30 years) to show the amount of the landscape in seedbank, < 1% cover, 1–10% cover, > 10–50% cover, > 50% cover, and converted. We used analysis of variance to calculate the variance in invaded area that could be attributed to the different uncertainties in model initialization and parameterization. We then used hierarchical partitioning^38^ to rank the importance of the main factors given that the interactions contributed little to the variation explained.

To investigate the impact of fire, we provided information to FARSITE at each time step, including fuel model associated with the current state for each cell, average weather conditions, and topography (described in detail in^29^).We calculated the amount of burned area at each time step, the number of years that have fire (while there is a maximum of one ignition per year, an ignition occurring in non-flammable vegetation will not result in a fire), and the number of fires that burned to the forest edge. We exported ST-Sim output to R to create all graphics and conduct statistical analyses using the R packages tidyverse^39^, raster^40^, and hier.part^41^ in R version 3.4.2^35^.

## Results

Model files and results are available as a U.S. Geological Survey data release^42^. See Supplementary 1 online for details on accessing the software and model.

### Model initialization

**Figure 2:**
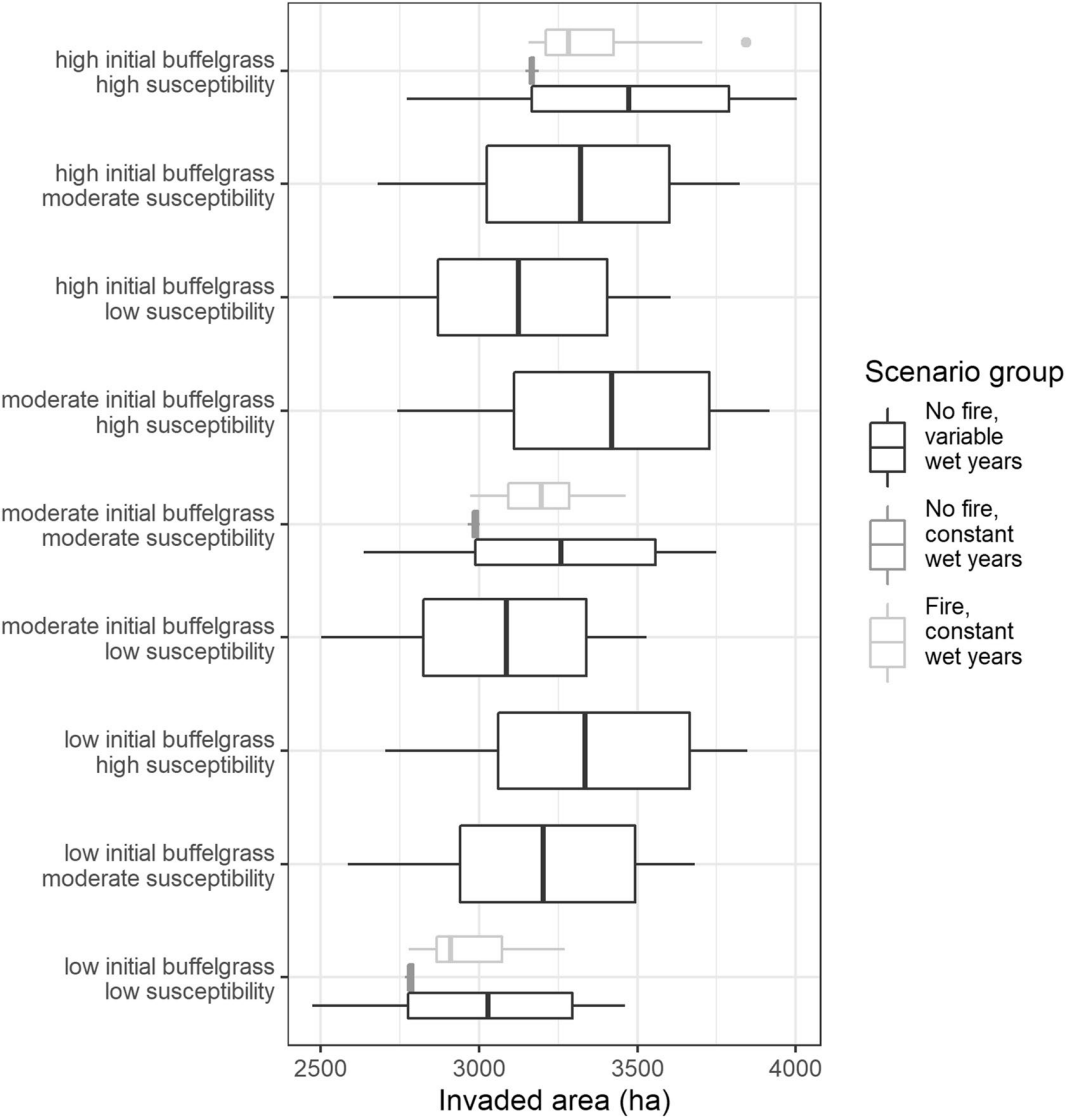
Variation in buffelgrass invaded area in Saguaro National Park (SAGU) at the end of 30 timesteps for initialization scenarios exploring sensitivity to amount of susceptible desert (susceptibility) and amount of buffelgrass initially on the landscape (initial conditions), combining levels of susceptibility and initial conditions for 20 Monte Carlo realizations for SAGU both with fire and without fire and with and without varying timing of wet years.

Sensitivity to model initialization, including the amount of desert susceptible to buffelgrass invasion and the initial amount of buffelgrass present on the landscape, did not result in major differences among runs (Fig. 2). Box plots displaying variation among invaded area for 20 Monte Carlo realizations of the nine simulations of various combinations of initialization all overlap for SAGU. This overlap exists even for simulations at both extremes of initialization—low initial buffelgrass and low suitability (low initialization) compared to high initial buffelgrass and high suitability (high initialization).

For the ‘no-fire, variable wet years’ scenario group, invaded area at the end of the simulation with low initialization had a mean of 3030 ha (ranging between 2473 ha and 3460 ha) and the simulation with high initialization had a mean of 3472 ha (ranging between 2772 ha and 4003 ha). Hierarchical partitioning identified susceptibility as a more important source of variation in invaded area at the end of the simulation than initial conditions (explaining 93% of the variation compared to 7%); the interaction between susceptibility and initial conditions was not significant and contributed minimally (Supplementary 1 Table S1 online). For the ‘no-fire, constant wet years’ scenario group, the boxplots exhibit minimal variation, indicating that most of the stochasticity in the no-fire simulations arose from wet-year timing. These boxplots did not overlap.

### Model parameterization

**Figure 3:**
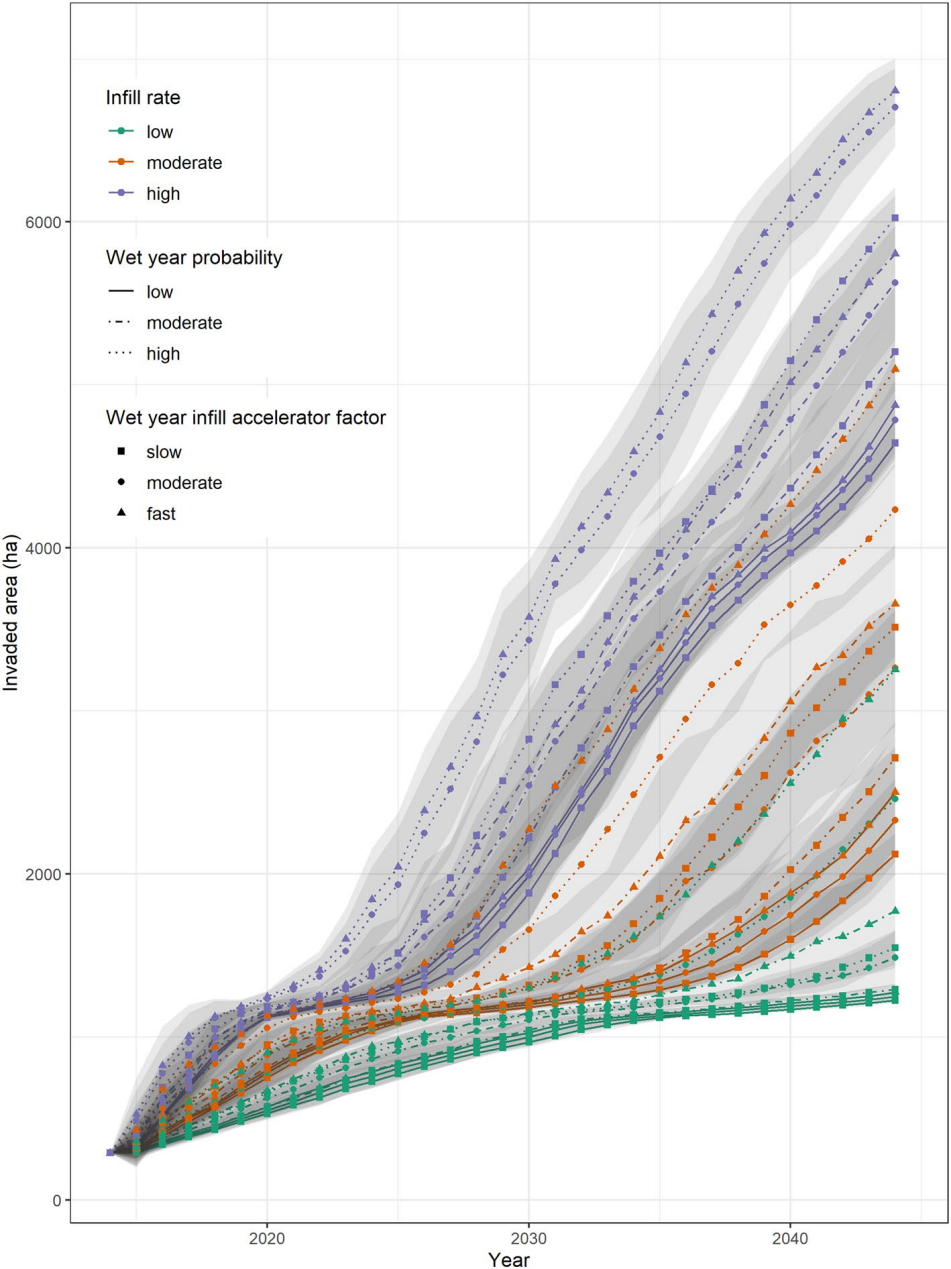
Area invaded by buffelgrass through time in Saguaro National Park, where area invaded is calculated by multiplying the area in each cover-class bin by the average cover within each bin, for 27 ecological scenarios combining uncertainty in the infill rates, the wet-year probability, and the wet-year infill acceleration factor. Grey area around lines display + /− one standard deviation.

Varying precipitation effects and infill rates resulted in large variations in the amount of invaded area at the end of the 30-year simulation (range of 1199 ha to 7134 ha; Fig. 3). Unlike sensitivity to initialization, the variation from simulation parameter values was much greater than the variation among Monte Carlo realizations (Fig. 3, grey area around lines displaying + /− one standard deviation). The infill rate had the greatest influence on area invaded at the end of the simulation according to hierarchical partitioning (78%), followed by wet-year probability (18%), with minimal influence from the wet-year infill acceleration factor (4%). The interaction between infill rate, wet-year probability, wet-year infill acceleration factor, and the three-way interaction were significant but contributed minimally compared to the direct effects (Supplementary 1 Table S2 online). Nine of the ten most invaded simulations were from scenarios that included the high infill rate. The scenario with a moderate infill rate but a high wet-year probability and a fast wet-year infill acceleration factor had a higher invaded area than scenarios with high infill rate but low wet-year probability. Infill rate combined with wet-year probability was more important than the wet-year infill acceleration factor, with all three wet-year infill acceleration factors generally clumped slow to fast within the wet-year probability and infill rate groupings.

**Figure 4:**
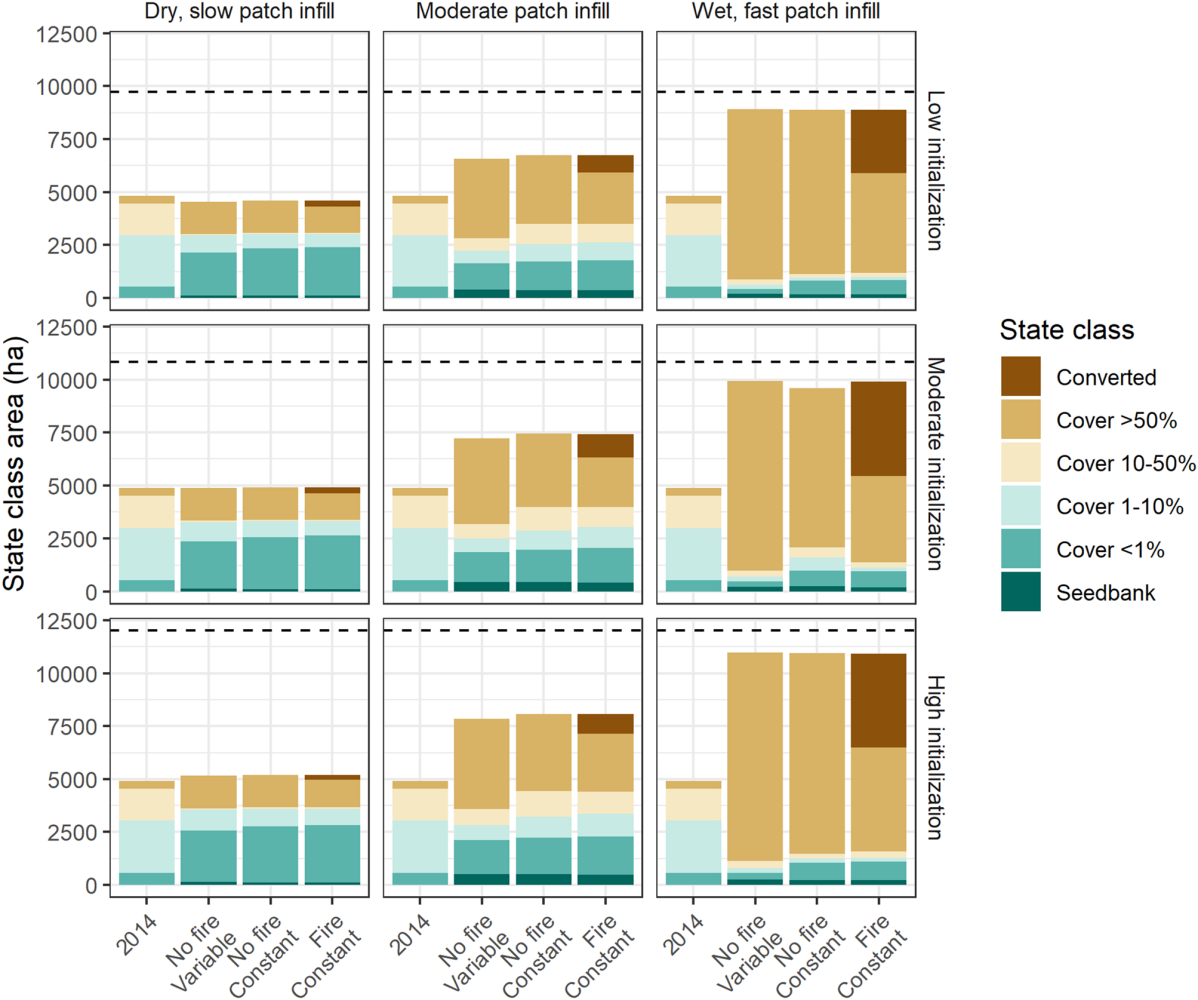
Area of landscape cells assigned to each buffelgrass cover class in Saguaro National Park at the start (2014) and end (2044) for three scenario groups. The scenario groups include simulations without fire run with both varying timing of wet years and constant timing of wet year, and simulations with fire run with constant timing of wet years. Facets show scenario results for the combination of three sets of ecological parameter values (dry-slow patch infill, moderate patch infill, and wet-fast patch infill) and three sets of initialization conditions. The dashed black line in each facet indicates the amount of desert ecosystem susceptible to buffelgrass invasion for that set of simulations.

There was a large shift towards the > 50% and converted cover classes in the wet-fast infill scenarios; this shift was greater on average for the variable wet-year timing simulations than the constant wet-year timing simulations (Fig. 4). By the end of the wet-fast infill simulations, buffelgrass has occupied most of the susceptible landscape, with invaded state classes occupying > 8800 ha in all scenario groups out of 9735 ha of susceptible area (low initialization) to > 10,600 ha out of 12,013 ha susceptible area (high initialization).

Variation in ecological uncertainties had more influence on invaded area at the end of the simulation than variation in model initialization within the nine scenarios that varied these two uncertainty groupings (98% contribution to variation compared to 2%). There was a significant interaction between the two uncertainty groupings, but it contributed little compared to the direct effects (Supplementary 1 Table S3 online).

### Influence of fire

Simulations with fire showed a similar pattern in the influence of uncertainty groups as simulations without fire (Fig. 4). The overall area of the landscape with some level of buffelgrass cover is the same for fire and no-fire runs, reflecting a model assumption that propagule dispersal for a converted patch is similar to that of a > 50% patch. There was overlap in the boxplots for invaded area after 30 years for the three initialization simulations that included fire (Fig. 2), indicating that initialization did not have a significant effect on model results for fire simulations. Fire simulations had constant wet-year timing; comparing fire simulations to the no-fire simulations with constant wet-year timing, fire simulations had much greater stochasticity than those without fire. However, comparing fire simulations to the no-fire simulations with variable wet-year timing, the stochasticity generated from fire appears to be less than the stochasticity generated by variability in wet-year timing. Overall, the stochasticity of the model resulted in greater variability in invaded area for simulations that included fire than simulations that excluded fire when wet-year timing was held constant (range across all scenarios of 4696 ha compared to 3690 ha; Supplementary 1 Figure S2 online), but variability in wet-year timing seemed to have a greater effect than fire. Ecological uncertainties had a greater influence than initialization uncertainties (Supplementary 1 Table S3 online), so we focus on ecological uncertainties to evaluate fire impacts on invaded area.

**Figure 5:**
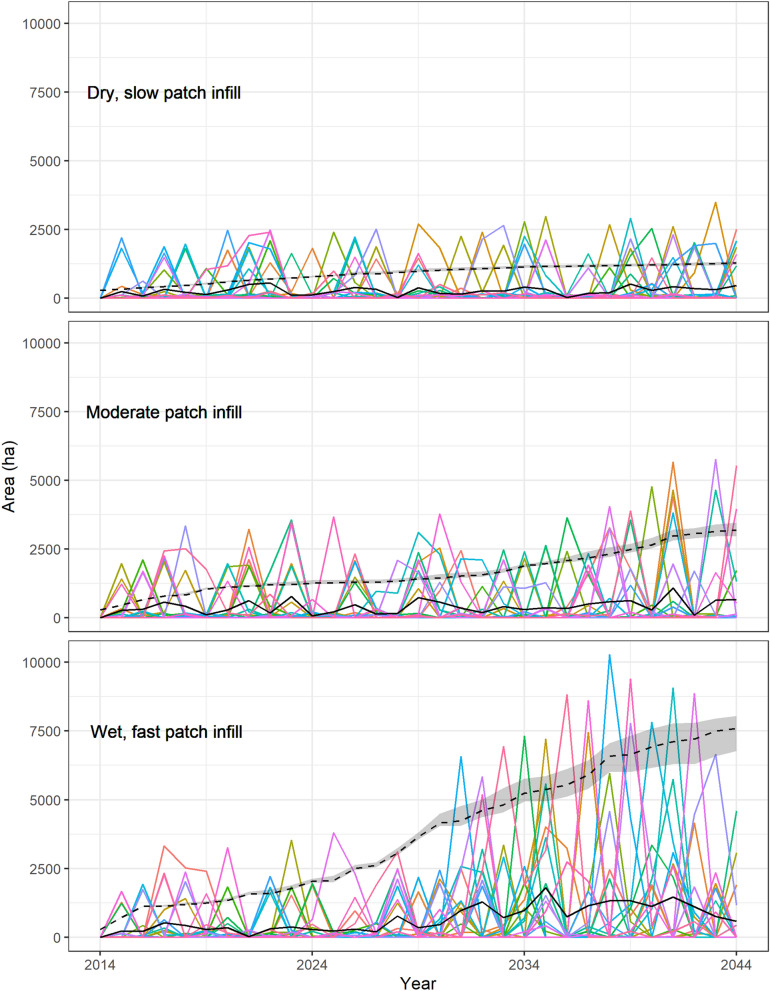
Area burned (colored lines represent different Monte Carlo realizations), the average area burned (solid black line), and the average area invaded through time (dashed black line) for 20 different Monte Carlo realizations for three different scenarios reflecting uncertain ecological conditions (low, moderate, and high infill rates, wet-year probabilities, and wet-year infill acceleration factors) in Saguaro National Park. Grey area around dashed line represents + /− 1 standard deviation.

There was large interannual variability in the amount of burned area (Fig. 5), and many years did not have a fire (Table 2). Generally, around half the years in the simulation had a fire, with a range of 7 to 22 years in the 30-year horizon having fires (Table 2). Years without fires resulted from both a lack of ignition and from “ignitions” occurring in unburnable locations. However, all scenarios had locations that burned multiple times, as seen in Table 2, where the cumulative area burned (sum across 30 years of annual burned area) was greater than landscape area burned (amount of the entire study area that burned in at least 1 year of the 30-year simulation). The size of fires (represented by the annual area burned) increased with increasing invaded area (Fig. 5; Figure S3) regardless of scenario. The average number of fires burning to the edge of the forest during the 30-year simulations ranged between 0 and 10 with an average around four (Table 2), compared to the number of fires occurring in the Rincon Mountain District containing the high elevation forests of 4 to 17 with an average of 11. Landscape burned area had a significant effect from the interaction between the two uncertainty groups (ecological uncertainty and initialization uncertainty), but initialization uncertainty by itself was not significant (Supplementary 1 Table S4 online). The cumulative area burned had a statistically significant main effect on patch infill rate (Supplementary 1 Table S5 online). Neither number of years with fires nor number of fires to forest edge had any significant predictors (Supplementary 1 Tables S6 and S7 online).

**Table 2:** Results from 30-year simulations for nine scenarios combining ecological uncertainties and initialization uncertainty for Saguaro National Park including the number of simulation years with a fire (defined as a year with an ignition and a burned area > 0 ha), cumulative burned area over the 30-year simulation, the amount of the entire study area that burned in at least one year of the 30-year simulation (landscape burned area), and the number of fires burning to the edge of the high-elevation forest (fires to forest).

Patch infill rate	Initialization	Number of years with fire	Cumulative burned area (ha)	Landscape burned area (ha)	Fires to forest edge
Dry-slow	Low	14.6 (11–20)	9142 (3684–14,149)	6788 (3582–8962)	3.95 (1–6)
Dry-slow	Moderate	15.2 (12–20)	8486 (3548–14,403)	6500 (3503–9922)	3.45 (0–7)
Dry-slow	High	14 (8–19)	8131 (1429–13,356)	5878 (1429–9500)	3.35 (0–6)
Moderate	Low	13 (7–16)	10,897 (2294–19,243)	7888 (2195–10,852)	3.85 (1–8)
Moderate	Moderate	14.1 (10–18)	12,397 (2850–20,213)	8427 (2799–12,180)	3.8 (0–8)
Moderate	High	13.8 (8–22)	13,437 (2589–26,687)	7912 (2596–11,307)	4.7 (1–9)
Wet-fast	Low	13.6 (11–17)	17,064 (6105–34,419)	10,897 (4619–15,423)	3.8 (0–10)
Wet-fast	Moderate	13.6 (10–20)	20,694 (9816–34,077)	13,317 (8696–16,951)	4.15 (2–8)
Wet-fast	High	14.6 (10–21)	22,596 (6593–41,774)	13,371 (6618–18,274)	4.45 (1–8)

All results are averaged across 20 Monte Carlo realizations with the range in parentheses.

## Discussion and conclusions

STSMs can be useful in identifying key uncertainties that may influence invasion forecasts. Future research on influential parameters could better inform decision making by providing more reliable predictions of the potential spread, infill, and impacts of invasive plants^15^. Many STSMs incorporate uncertainty into models by providing a distribution of values for uncertain parameters that the model can draw from across Monte Carlo realizations (e.g.^14,43,44^). By running many Monte Carlo realizations, uncertainty around state class distribution and transition amounts at the end of the simulation is provided. We chose instead to run multiple Monte Carlo realizations of scenarios with different combinations of these uncertain values to answer the question of which parameter values have the greatest influence on invaded area and cover-class distribution to allow statistical evaluation of the influence of the different parameters. Thus, we have provided information about which ecological parameter uncertainties are important to target with research to thereby better inform management decisions.

We refined previous research results aimed at forecasting the scope of the buffelgrass problem in Saguaro National Park by evaluating sensitivity to uncertainties in the model. Our sensitivity analyses showed that uncertainty in model initialization (habitat suitability) had less influence on results than uncertain ecological parameters (infill rates and wet-year probability, timing, and effect). Examining the contribution of components of ecological uncertainty, we found that uncertainty in infill rates had the greatest influence on future landscape conditions, including the amount of buffelgrass on the landscape and the size and frequency of fires; the variation arising from different infill rate values was greater than the variation arising from stochasticity. These results highlight the potential need to better understand the temporal dynamics associated with increases in buffelgrass abundance over knowing the probability of wet years or the effects of wet-year infill acceleration factor.

The rate of buffelgrass infill is difficult to quantify because of the high variability in precipitation in the region. Olsson et al.^45^ reported relatively constant increase in infestation area from 1980 to 2008 based on interpretation of aerial photographs for 11 sites, but there was variability between sites. Another study in the Arizona Sonoran Desert found variable patch expansion over time^46^. Neither study showed a relationship between growth and precipitation, but the first study did not include annual time steps and the second only collected 3 years of data; further, both studies were within relatively homogenous climate areas whereas Saguaro National Park encompasses greater variability in climate conditions. Results may also be influenced by the spotty nature of precipitation where weather data from a nearby climate station may not represent the weather experienced by the buffelgrass patch. Additionally, other meteorological variables have important, but not fully understood, impacts on buffelgrass growth and reproduction, including precipitation duration, humidity, wind, cloud cover, and temperature^47,48^. Precipitation in the prior years is also important in determining tiller and biomass production in perennial warm-season grasses in desert grasslands^49^.

Expert elicited observations suggest there is a relationship between buffelgrass infilling and precipitation^29^. While annual precipitation in the region is expected to decline, there is still great uncertainty in precipitation forecasts for the region from known issues with precipitation predictions^50^. Recent analyses of future climate predictions for the region do lean towards a reduction in monsoon season precipitation^51–53^, suggesting that landscape outcomes associated with our dry-slow and moderate scenarios may be closer to future conditions than the wet-fast patch infill scenarios. However, increasing temperatures in winter^54^ may result in increased germination and seedling survival in non-monsoon seasons, and temperature projections are less problematic than precipitation.

A related invasive, wind dispersed perennial C4 grass, *Cenchrus setaceus* (Forssk.) Morrone (fountain grass)*,* showed a strong relationship between precipitation and total seed production^55^ and precipitation and fecundity^56^. These factors, related to propagule pressure, can be expected to contribute to the observed effect of precipitation on buffelgrass infilling rate. In the eastern United States, propagule rain and disturbance were the most important predictors of changes in abundance of three different invasive plant species^57^. Buffelgrass also exhibits a strong growth response to precipitation^31^. Future research exploring buffelgrass infill rates, and the influence of precipitation on this rate, may decrease uncertainty in model simulations.

Invasive species’ impacts vary widely, and the grass-fire cycle is a well-studied case of invasive grasses altering the scale, frequency, and/or intensity of wildfires while also promoting further invasion^58–60^. Buffelgrass can transform landscapes in the Arizona Sonoran Desert in the absence of fire^25^, with transformation occurring in the Santa Catalina Mountains within 20 years of invasion. Buffelgrass can also increase both the scale and frequency of fire thereby affecting non-fire adapted flora and fauna in the desert ecosystem^21,22,26,28^. Our simulation results reflect the combination of these two processes, with a similar pattern of increasing invasion over time but with faster invasion with the inclusion of fire, particularly in the wet-fast scenarios. SAGU staff were concerned about the number of fires that could burn to the forest edge as these could potentially lead to much larger fires and modify the frequency of fires currently observed in the forest ecosystem, and historically this fire pattern has not existed. The simulations showed that this pattern could potentially be an issue in the future, with the desert ecosystem carrying fire to the forest.

Managers need information to support decision making despite the uncertainty surrounding future conditions, particularly forecasts of “tipping points”^61^ such as the converted state in our simulations. Regardless of uncertainties, all scenarios had increasing buffelgrass cover at the end of the simulation, indicating that buffelgrass may invade a larger portion of the study area than that predicted in the final time step of the simulations. These commonalities across scenarios without management activities allow assessment of the potential scale of the buffelgrass problem in Saguaro National Park, addressing a second question that STSMs can be useful for in invasion contexts^15^. We found that scenarios spanning ranges of uncertainty related to buffelgrass infill rates all predicted increasing buffelgrass cover in the absence of management actions over a 30-year time horizon. Further, these parameters were more influential to model outcomes than initial conditions, and variation in the base infill rates had the greatest influence on the amount of buffelgrass on the landscape and the size and frequency of fires. Given the substantial differences in the predicted amount of buffelgrass on the landscape at the end of our simulations and the size and frequency of fires for dry-slow scenarios compared to wet-fast scenarios, additional research on the base infill rates of buffelgrass could greatly reduce uncertainty in model simulations and improve model performance. Our approach could be widely applied to buffelgrass in other locations where it is invading and where it could potentially impact fire cycles; answers to the research questions we have listed would inform these efforts. The approach could be used for other species as well, to help inform the widespread issue of invasive grasses and their impacts to the fire cycle.

## Supplementary information

Supplementary Information.
