# Supplementary material for: Assessing ecological uncertainty and simulation model sensitivity to evaluate an invasive plant species’ potential impacts to the landscape
Source: Sci Rep. 2020 Nov 4;10:19069. doi: 10.1038/s41598-020-75325-w (PMC7643150; doi:10.1038/s41598-020-75325-w)
Supplement: Supplementary file 1 — Supplementary Information. [file 41598_2020_75325_MOESM1_ESM.pdf]

Supplementary material

Title: Assessing ecological uncertainty and simulation model sensitivity to evaluate an invasive plant species' potential impacts to the landscape

Authors: Catherine S Jarnevich, Nicholas E Young, Catherine Cullinane Thomas, Perry Grissom, Dana Backer, Leonardo Frid

Supplementary 1. This appendix includes additional results, both tabular and spatial, from the state and transition simulation model (STSM) scenarios developed for Saguaro National Park to explore the impact of ecological uncertainty and sensitivity of the model to parameterization.

All simulations described in the paper were run using the SyncroSim software (version 2.0.41) including the ST-Sim module (version 3.1.21) and the farsite-stsim package (version 3.1.21); versions of the software exist for Windows (64-bit Windows 7 or higher) and Linux (running Mono). However, FARSITE simulations require Windows. The following steps are required to run this model in ST-Sim:

1. Download and install the SyncroSim (with ST-Sim) software **version 2.0.41** , available at: https://syncrosim.com/downld-prev-versions/
2. Download the stsim-farsite package (version 3.1.21) from https://github.com/ApexRMS/stsim-farsite/releases.
3. Add the stsim-farsite package to SyncroSim under "File | Packages"
4. If you plan to run simulations with this version stsim-farsite you will also need to install FARSITE 4.1.055 and QGIS 2.4.0.
5. Download and unzip the case study ST-Sim model files from the Science Base Repository at (DOI link to ScienceBase repository will be inserted upon article acceptance).

6. Use the SyncroSim software to open the library file called STSM Buffelgrass.ssim

Table S1. ANOVA results for buffelgrass invaded area at the end of a 30-year simulation for two different model initialization factors (Susceptibility = amount of the landscape defined as susceptible to invasion; Initial conditions = amount of buffelgrass on the landscape at the start of the simulations), each with three levels (low, moderate, high susceptibility and initial conditions). Test: In the evaluation of sensitivity to landscape starting states, is there an interaction between modeler decisions on landscape susceptibility to buffelgrass invasions and where and how much buffelgrass is on the landscape (initial conditions)? Result: Susceptibility is the only factor with significant differences in the amount of buffelgrass invaded area.

	df	SS	Mean Sq	F value	Pr(>F)
Susceptibility	2	2,709,169	1,354,584	10.935	<0.001
Initial conditions	2	200,970	100,485	0.811	0.45
Susceptibility: Initial conditions	4	67,972	16,993	0.137	0.97
Residuals	171	21,182,221	123,873		

Table S2. ANOVA results for buffelgrass invaded area for Saguaro National Park at the end of a 30-year simulation for three different model parameters affecting buffelgrass patch infill rates (Infill rate, Accelerator = Wet-year infill acceleration factor; probability = wet-year probability), each with three levels. Test: In the evaluation of sensitivity factors affecting cover-class transition rates, is there an interaction between any of the three? Result: The interaction between infill rate, wet-year probability, wet-year infill acceleration factor, and the three-way interaction were significant but contributed minimally compared to the direct effects.

	df	SS	Mean Sq	F value	Pr(>F)
Acceleration factor	2	51,009,076	25,504,538	354.9	<0.001
Probability	2	241,458,836	120,729,418	1,680.1	<0.001
Infill rate	2	1,373,814,700	686,907,350	9,559.1	<0.001
Acceleration factor: Probability	4	19,715,486	4,928,871	68.6	<0.001
Acceleration factor: Infill rate	4	3,329,714	832,428	11.6	<0.001
Probability: Infill rate	4	11,393,658	2,848,414	39.6	<0.001
Acceleration factor: Probability: Infill rate	8	4,523,811	565,476	7.9	<0.001
Residuals	513	36,863,621	71,859		

Table S3. ANOVA results for buffelgrass invaded area at the end of a 30-year simulation that included fire transitions for two different classes of uncertainty including three levels of initialization (combination of amount of the landscape defined as susceptible to invasion and amount of buffelgrass on the landscape at the start of the simulations) and three levels of patch infill rates (combination of wet-year infill acceleration factor, wet-year probability, and infill rate). Test: When considering uncertainty related to initialization and cover-class transition rates, is there an interaction between the two? Result: There was a significant interaction between the two uncertainty groupings, but it contributed little compared to the direct effects.

	df	SS	Mean Sq	F value	Pr(>F)
Initialization	2	7,970,461	3,985,230	72.47	<0.001
Cover-class transition rates	2	950,958,068	475,479,034	8,646.43	<0.001
Initialization: Cover-class transition rates	4	8,745,091	2,186,273	39.76	<0.001
Residuals	171	9,403,521	54,991		

Table S4. ANOVA results for landscape burned area during a 30-year simulation for two different classes of uncertainty including three levels of initialization (combination of amount of the landscape defined as susceptible to invasion and amount of buffelgrass on the landscape at the start of the simulations) and three levels of patch infill rates (combination of wet-year infill acceleration factor, wet-year probability, and infill rate). Test: When considering uncertainty related to initialization and cover-class transition rates, is there an interaction between the two related to burned area? Result: The interaction is significant at the 0.05 level.

	df	Sum Sq	Mean Sq	F value	Pr(>F)
Cover-class transition rates	2	1,214,663,960	606,847,153	108.9	< 0.001
Initialization	2	24,061,058	11,880,599	2.1	0.12
Initialization: Cover-class transition rates	4	68,244,339	17,061,085	3.1	0.01
Residuals	171		5,572,914		

Table S5. ANOVA results for cumulative area burned during a 30-year simulation for two different classes of uncertainty including three levels of initialization (combination of amount of the landscape defined as susceptible to invasion and amount of buffelgrass on the landscape at the start of the simulations) and three levels of patch infill rates (combination of wet-year infill acceleration factor, wet-year probability, and infill rate). Test: When considering uncertainty related to initialization and cover-class transition rates, is there an interaction between the two related to cumulative burned area? Result: Only cover-class transition rates are significantly different.

	df	Sum Sq	Mean Sq	F value	Pr(>F)
Cover-class transition rates	2	4197377957	2098688979	66.138	<0.001
Initialization	2	149351176	74675588	2.353	0.098
Initialization: Cover-class transition rates	4	221472709	55368177	1.745	0.14
Residuals	171	5426194571	31732132		

Table S6. ANOVA results for number of years with fire during a 30-year simulation for two different classes of uncertainty including two levels of initialization (combination of amount of the landscape defined as susceptible to invasion and amount of buffelgrass on the landscape at the start of the simulations) and three levels of patch infill rates (combination of wet-year infill acceleration factor, wet-year probability, and infill rate). Test: When considering uncertainty related to initialization and cover-class transition rates, is there an interaction between the two related to number of years with fire? Result: None were significantly different.

	Df	SS	Mean Sq	F value	Pr(>F)
Cover-class transition rates	2	11.7	5.9	1.6	0.213
Initialization	2	4.6	2.3	0.61	0.55
Initialization: Cover-class transition rates	4	14	3.5	0.93	0.45
Residuals	171	643.1	3.8		

Table S7. ANOVA results for number of fires burning to forest edge during a 30-year simulation for two different classes of uncertainty including three levels of initialization (combination of amount of the landscape defined as susceptible to invasion and amount of buffelgrass on the landscape at the start of the simulations) and three levels of patch infill rates (combination of wet-year infill acceleration factor, wet-year probability, and infill rate). Test: When considering uncertainty related to initialization and cover-class transition rates, is there an interaction between the two related to number of fires burning to the forest edge? Result: None were significantly different.

	df	Sum Sq	Mean Sq	F value	Pr(>F)
Cover-class transition rates	2	4.6	2.3	0.6	0.55
Initialization	2	11.7	5.9	1.6	0.21
Initialization: Cover-class transition rates	4	14	3.5	0.9	0.45
Residuals	171	643.1	3.8		

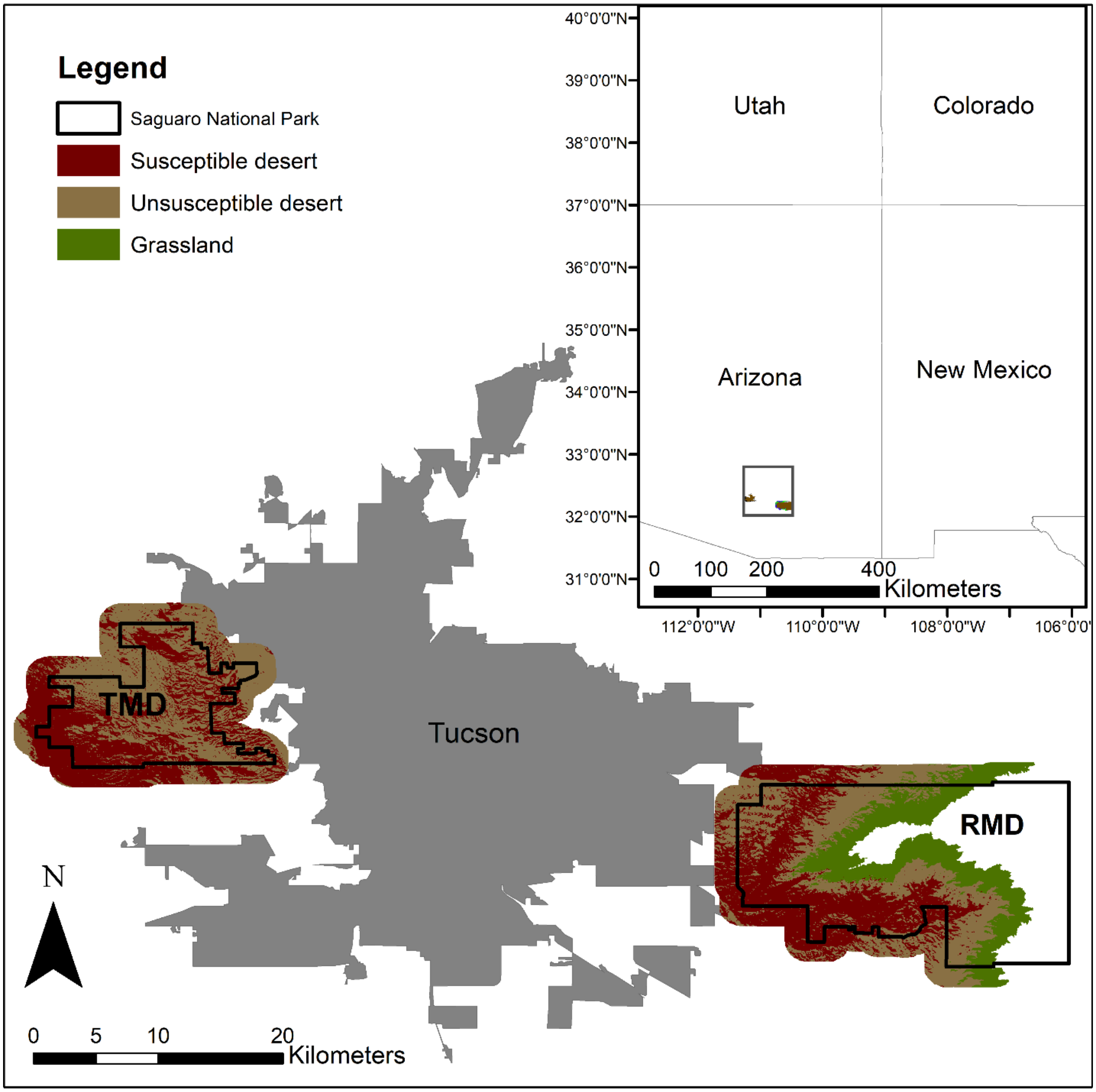

Figure S1. Saguaro National Park, located in Southern Arizona, USA, is divided into two different units on either side of Tucson including the Tucson Mountain District (TMD) and Rincon Mountain District (RMD). Map uses the geographic coordinate system and were built using Esri ArcGIS 10.5 (www.esri.com/sofware/arcgis).

a)

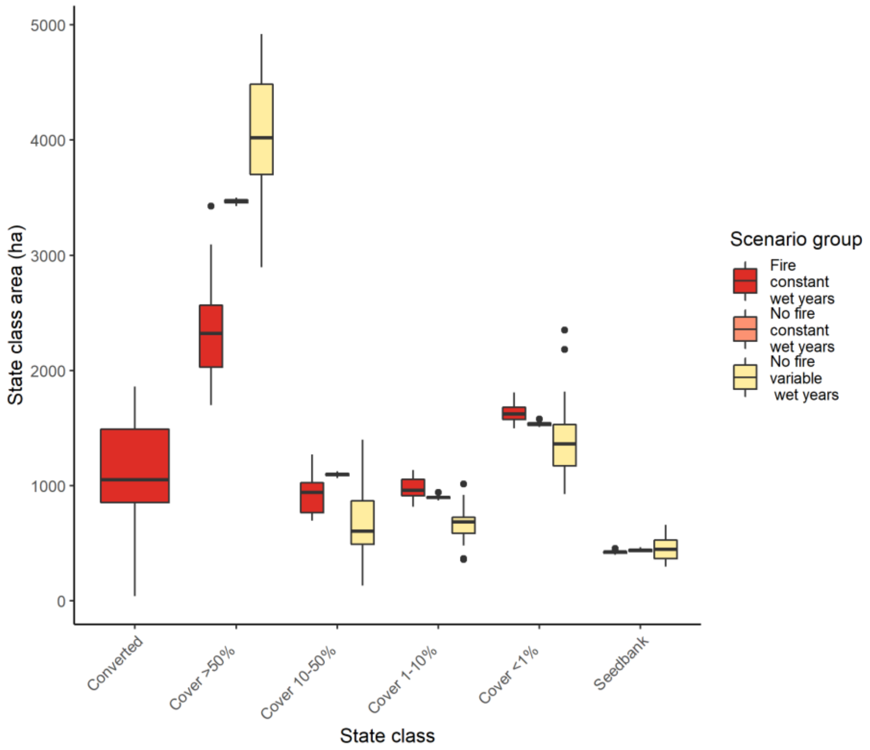

State class area (ha)
5000
4000
3000
2000
1000
0
Converted
Cover >50%
Cover 10-50%
Cover 1-10%
Cover <1%
Seedbank
State class
Scenario group
Fire constant wet years
No fire constant wet years
No fire variable wet years

b)

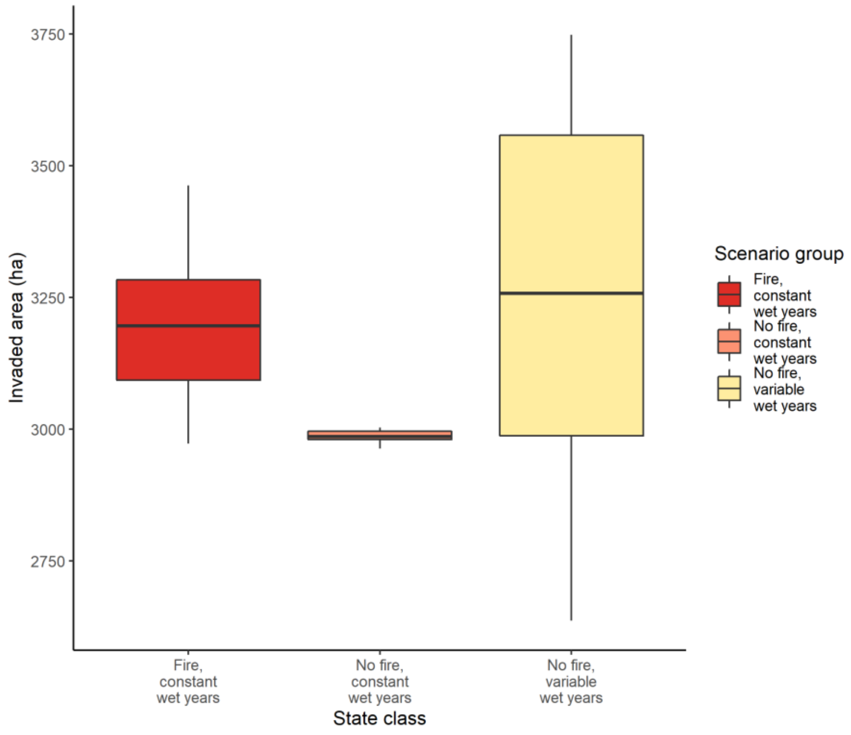

Figure S2. For the moderate conditions scenario only (center plot of figure 4), a) a boxplot highlighting the variation among iterations in state class area by state class and b) a boxplot of total invaded area where invaded area is calculated by multiplying the area covered by the state class by the mean cover for that state class (e.g., >50% multiplied by 0.75). Single points in (a) are outliers.

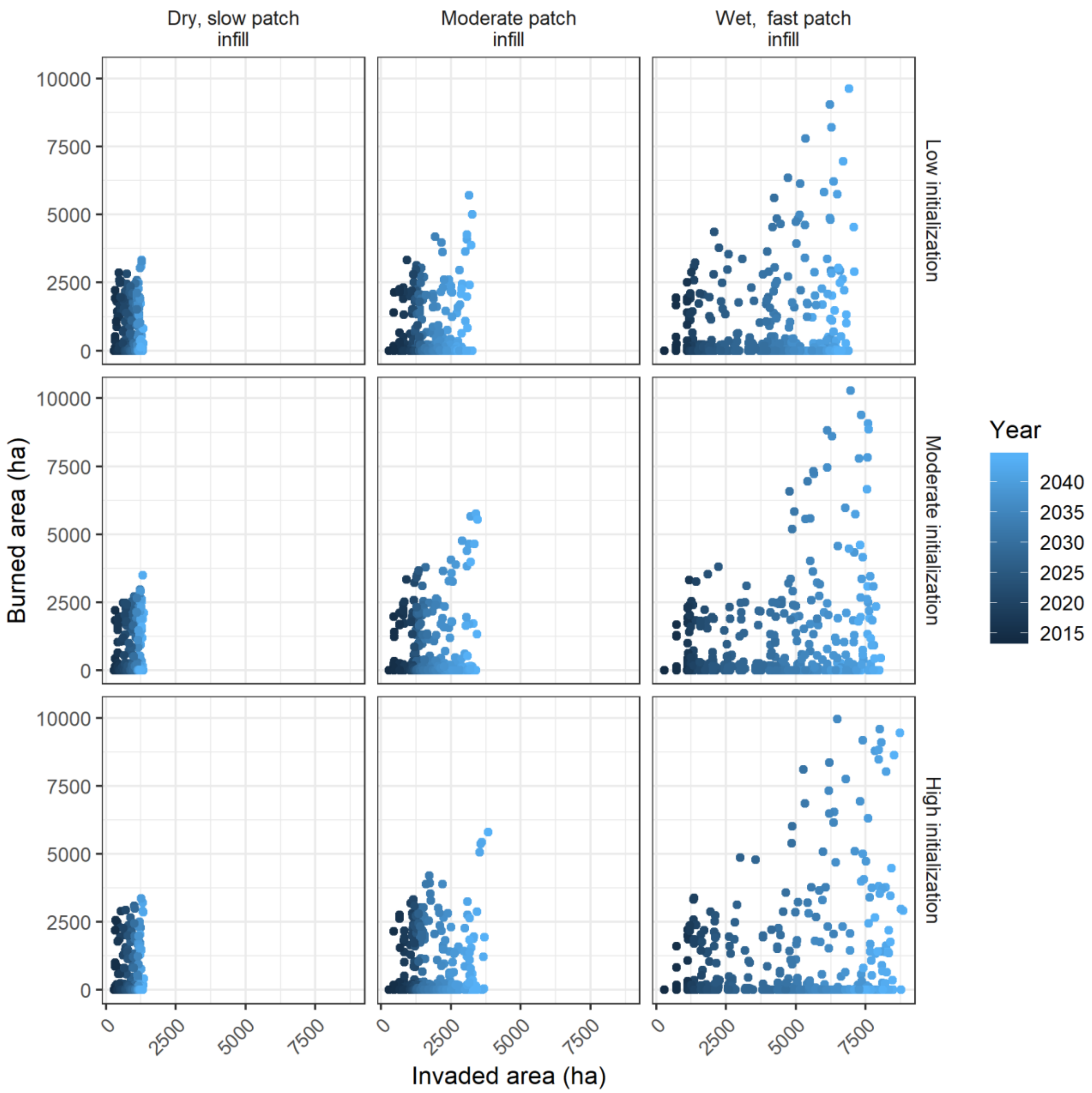

Figure S3. The relationship between area invaded (ha) by buffelgrass through time, where area invaded is calculated by multiplying the mean of the cover-class bin by the area classified as the cover-class bin, and burned area (ha) colored by the simulation timestep (2014 to 2044) with 20 points per time step resulting from the 20 Monte Carlo realizations for Saguaro National Park.
